# A rapid and effective approach to building a life-saving multidisciplinary team for transferred postpartum haemorrhage patients: leveraging trauma experience—a retrospective study

**DOI:** 10.1186/s12884-025-07204-z

**Published:** 2025-02-11

**Authors:** Pei-Hsiu Yu, Kuo-Shu Hung, Lin Kang, Tsung-Han Yang, Chun-Hsien Wu, Pei-Yin Tsai, Chih-Jung Wang, Yi-Ting Yen, Chen-Hsiang Yu, Chiung-Hsin Chang

**Affiliations:** 1 Department of Obstetrics and Gynecology, National Cheng Kung University Hospital, College of Medicine, National Cheng Kung University, Tainan, Taiwan; 2 Department of Obstetrics and Gynecology, AnAn Women and Children Clinic, Tainan, Taiwan; 3 Department of Surgery, National Cheng Kung University Hospital, College of Medicine, National Cheng Kung University, Hospital, Tainan, Taiwan; 4 Department of Obstetrics and Gynecology, Kuo General Hospital, Tainan, Taiwan

**Keywords:** Postpartum haemorrhage, Multidisciplinary team, Trauma team, Patient transfer, Blood transfusion, Team training

## Abstract

**Background:**

Establishing an efficient multidisciplinary team for transferred postpartum haemorrhage (PPH) cases is challenging due to limited clinical exposure. We hypothesised that leveraging trauma team experience could effectively facilitate the development of such a team within a short timeframe.

**Methods:**

In September 2019, a multidisciplinary team was established at our tertiary care centre to provide rapid management of critical PPH cases transferred from the obstetric clinic, prioritising immediate resuscitation and haemostatic interventions. This historical cohort study (2017–2022) compared outcomes before (2017–2018, before group [BG]) and after (2019–2022, after group [AG]) team establishment. Outcomes included process-related quality indicators, clinical measures such as length of hospital stay, intensive care unit (ICU) days, presence of the lethal triad, and hysterectomy rate.

**Results:**

Of the 71 PPH patients transferred during the study period, 24 were in the BG and 47 in the AG. The AG demonstrated higher use of tranexamic acid (33.33% vs. 74.47%, *P* = 0.002), shorter time to the first blood transfusion (11 vs. 8 min, *P* = 0.029), and increased rates of arrival in the operating room within 60 min (25% vs. 80%, *P* = 0.014). Clinical outcomes showed reduced rates of cardiopulmonary resuscitation (16.67% vs. 0%, *P* = 0.011) and shorter ICU stays (4 vs. 1 day, *P* = 0.005) in the AG.

**Conclusions:**

Leveraging trauma team expertise is an effective strategy for establishing a multidisciplinary PPH team, significantly improving outcomes for critically ill PPH patients transferred from obstetric clinics.

## Background

Postpartum haemorrhage (PPH) is a leading cause of maternal morbidity and mortality, accounting for 27% of maternal deaths globally and 13.7% of maternal death in the United States [1, 2]. In Taiwan, PPH accounts for 30% of maternal deaths, 39% of maternal morbidities, and 69% of peripartum hysterectomies [3]. PPH management is particularly challenging in Taiwan because approximately 28% of births occur in small obstetric clinics, where immediate access to comprehensive emergency care is often unavailable [4]. Similar challenges are evident in Japan, where recent studies indicate that nearly half of maternal deaths due to obstetric haemorrhage were potentially preventable with timely and appropriate interventions [5].

Timely interventions are essential to prevent bleeding-related deaths [2, 6, 7]. However, managing multiple tasks within constrained timeframes is challenging and prone to medical errors. Establishing a multidisciplinary rapid response team is a crucial step in addressing these issues [8–13]. Nevertheless, the formation of such teams does not guarantee immediate improvements in clinical outcomes because it takes additional training and team-building to develop team proficiency [14, 15]. Simulation is the most widely adopted method to enhance team competency. However, despite its extensive use, few studies have directly linked simulation training to improved clinical outcomes in PPH patients [16–18]. Managing critical bleeding patients requires seamless cooperation among surgeons, anaesthesiologists, operating room (OR) staff, blood bank personnel, emergency department physicians, interventional radiologists, and nurses. Constructing an effective multidisciplinary team is complex, and its processes have not been extensively studied in the literature.

Trauma surgeons have extensive frontline exposure to patients with critical bleeding. They possess valuable expertise in resuscitation, massive bleeding management, damage control strategies, and team coordination. Moreover, trauma surgeons are adept at identifying deficiencies in patient management systems and protocols, thereby enhancing outcomes [19–21]. Leveraging the expertise of trauma surgeons could facilitate the development of an effective rapid response team for PPH [22–24]. However, few studies have explored this approach.

In 2019, we successfully implemented a PPH team activation system through collaboration between trauma surgeons and obstetricians. This study examined the effect of the multidisciplinary PPH team, adapted from the trauma team model, on the management and outcomes of patients.

## Materials and methods

### Study population

This retrospective cohort study investigated patients with PPH who were transferred from regional clinics to the emergency room (ER) of our hospital, a tertiary medical centre, between 1 January 2017 and 31 December 2022. This study was approved by the Institutional Review Board of National Cheng Kung University Hospital, and the requirement for informed consent was waived.

PPH was defined as with cumulative blood loss of more than 1000 ml or blood loss accompanied by signs or symptoms of hypovolemia within 24 h after birth regardless route of delivery. Patients with PPH experiencing haemorrhage within 24 h after delivery were included. Those with secondary PPH or missing clinical data were excluded. Data collected from electronic medical records included demographic characteristics, vital signs, time of ER arrival, time of blood transfusion, total blood transfusion volume, details of haemostatic management (i.e., surgery, transcatheter arterial embolization [TAE], or conservative treatment), and laboratory test results.

This historical cohort study (2017–2022) compared patient outcomes before (2017–2018, before group [BG]) and after (2019–2022, after group [AG]) the establishment of the multidisciplinary team.

### Process-related quality indicators

Since the primary purpose of establishing the multidisciplinary team was to enhance patient care efficiency, we prioritized evaluating the team’s operational performance over clinical outcomes. To objectively measure the team’s efficiency in managing critical postpartum haemorrhage (PPH), we selected process-related quality indicators as outcomes. These indicators assessed two essential dimensions of patient care:

The first dimension focused on rapid resuscitation and haemostasis, evaluated by measuring the time to first transfusion, time to initial intervention, time to operating room (OR), and time to angiography room. The second dimension addressed adequate resuscitation to prevent coagulopathy, assessed through the administration of fresh frozen plasma (FFP) within the first hour and the use of tranexamic acid in the emergency room (ER).

### Clinical outcomes

The clinical outcomes included the duration of hospitalization, length of ICU stay, occurrence of cardiopulmonary resuscitation (CPR) during resuscitation, mortality rate, hysterectomy rate, and the presence of the lethal triad. The lethal triad, defined as the concurrent presentation of coagulopathy, acidosis, and hypothermia, is a critical complication resulting from persistent shock and massive transfusion in bleeding patients. The presence of the lethal triad is associated with a high risk of mortality. High-quality resuscitation and timely haemostasis are essential to reducing this risk. Coagulopathy was defined as prothrombin time (PT), expressed as the international normalised ratio (INR) > 1.5.

### Translating trauma team experience to PPH management: development of a multidisciplinary PPH team

In 2010, NCKUH established a trauma team with extensive experience in managing patients with critical bleeding. This team developed an efficient workflow involving coordination between ER physicians, nurses, anaesthesiologists, OR nurses, radiologists, and blood bank personnel, facilitating a rapid response to trauma cases. This collaborative approach ensured clear communication and a shared understanding of principles among all team members. Additionally, the team implemented strategies to optimise blood transfusions, such as the development of a tailored massive transfusion protocol aligned with the hospital’s system. These strategies enabled prompt and effective blood transfusion for patients with bleeding, minimising the risk of complications associated with the lethal triad.

To enhance the quality of care for transferred patients with PPH at our hospital, a multidisciplinary rapid response team was initiated in October 2018. Trauma surgeons shared their established team experience and protocol development expertise with obstetricians to create a dedicated multidisciplinary team for critical PPH cases. During the preparation phase, trauma surgeons, while not direct members of the PPH team, provided crucial guidance in team organization and coordination. They helped integrate key team members - including anesthesiologists, ER physicians, OR nurses, and blood bank personnel - and collaborated with obstetricians to develop rapid transfusion protocols aimed at preventing the lethal triad. Building on this trauma team experience and guidance, the multidisciplinary PPH team was established and became fully operational within three months, officially commencing its services in February 2019.

The multidisciplinary team comprised senior and junior obstetricians, ER physicians, nurses, anaesthesiologists, radiologists, ICU specialists, blood bank personnel, nursing staff, and support staff. Activation criteria for the multidisciplinary team were defined to focus on patients with PPH and unstable vital signs, specifically a heart rate exceeding 120 bpm and systolic blood pressure less than 90 mmHg, who were planned for transfer to our ER.

**Fig. 1:**
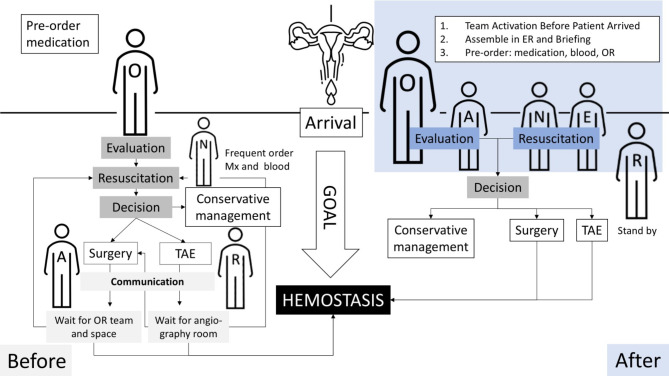
Team and Resource Preparation Before and After Establishment of a Multidisciplinary Team. The major differences are highlighted in the blue shaded areas. After Implementation: Team activation via text alerts all members. Key personnel assemble in the ER for briefing, enabling simultaneous resuscitation, decision-making, and operating room (OR) preparation. This parallel processing is critical for unstable patients requiring emergency surgery, reducing delays caused by sequential communication. Before Implementation: Only obstetricians (O) and ER nurses awaited patient arrival. Other specialists (A, E, R) were contacted post-evaluation, causing potential delays in critical interventions. Resource Preparation: After Implementation: Blood products, medications, and equipment for massive transfusion are prepared proactively before patient arrival. Before Implementation: Resources were mobilized reactively, often delaying care during emergencies. (Abbreviations: O, Obstetricians; A, Anaesthesiologist; N, Nursing staff; E, Emergency department physician; R, Interventional radiologist; OR, Operating room; TAE, Transcatheter arterial embolization.)

The patient management protocol before and after the establishment of the multidisciplinary PPH team is illustrated in Fig. 1.

### PPH management before establishing protocol

Before the establishment of the multidisciplinary PPH team (left column, Fig. 1), the obstetrician and ER nurses would remain on standby in the ER, awaiting the patient’s arrival. They independently evaluated the patient, initiated resuscitation measures, prescribed medications, and ordered laboratory examinations. If a patient required a procedure, such as intubation or the placement of a large-bore venous catheter, the anaesthesiologist or ER physician collaborated with the obstetrician and performed the procedure upon request. If additional interventions were required after the initial resuscitation, the obstetricians communicated with the anaesthesiologist and the OR or radiology department to prepare for the patient to receive the necessary care.

### PPH management after establishing protocol

After the multidisciplinary team was established (right column, Fig. 1), all team members received a text message notification upon team activation. This notification included obstetricians, ER physicians, nurses, anaesthesiologists, radiologists, ICU specialists, blood bank personnel, and support staff. Before the patient’s arrival, the obstetricians, ER physicians, nurses, anaesthesiologists, and support staff assembled in the ER. The obstetricians briefed the team on the patient’s condition and discussed potential strategies. To minimise the burden of tasks during critical situations, the team completed essential preparations before the patient’s arrival in the ER. These preparations included ordering type O red blood cells (RBC), securing one gram of tranexamic acid, preparing a rapid infusion system, and setting up a large-bore central venous catheter kit.

Upon the patient’s arrival, the ER physician initiated resuscitation while the obstetricians concurrently assessed the source of bleeding and formulated treatment strategies. The obstetricians assumed a leadership role, coordinating with the multidisciplinary team. The anaesthesiologist ensured the availability of an OR, and the OR nurses remained on standby for surgery until it was confirmed that surgery was unnecessary. Through this coordinated effort between ER physicians, anaesthesiologists, and obstetricians, the multidisciplinary team effectively managed multiple time-sensitive tasks simultaneously for critically ill patients. This approach enabled the obstetricians to focus on evaluation and decision-making, with reduced involvement in resuscitation and repeated communication, thus allowing them to concentrate on patient evaluation and treatment.

### Definition of conservative treatment

Conservative management was defined as a strategy in which the patient did not undergo surgery under anaesthesia or TAE within the first six hours of arrival. This approach encompassed various interventions, including blood transfusions, medical treatments, intrauterine balloon compression, sandbag compression, and bedside removal of retained placenta.

### TAE indication

TAE is a procedure performed by an interventional radiologist to embolize, or block, an active source of arterial bleeding, effectively controlling haemorrhage. TAE was performed when bleeding remained refractory to initial conservative management. The location of the TAE procedure was determined by clinical conditions. For cases without surgical indications, TAE was conducted in the angiography suite with continuous haemodynamic monitoring and blood product support. When surgical indications were present, TAE was performed in the OR post-surgery under the supervision of an anaesthesiologist, with ongoing resuscitation to ensure patient safety.

### Surgical indication

Surgical treatment indications for PPH included haemodynamic instability, retained placental tissue confirmed by ultrasound, significant hemoperitoneum, and obvious genital tract laceration.

### Salvage procedure

A salvage procedure refers to additional interventions implemented after the initial haemostatic intervention when the patient experiences persistent or recurrent bleeding. These interventions may include procedures such as TAE or surgery.

### Subgroup analyses

Patients requiring more than six units of packed red blood cells (pRBC) transfusion within 24 h were classified as the significant bleeding subgroup. Subgroup analysis was conducted to evaluate the impact of multidisciplinary team intervention in this high-risk population.

### Statistical analysis

Differences in categorical variables between groups were compared using *χ*^2^ analysis or Fisher’s exact test, whereas differences in continuous variables were examined using the Mann–Whitney U test. Statistical significance was defined as *P* < 0.05.

## Results

**Fig. 2:**
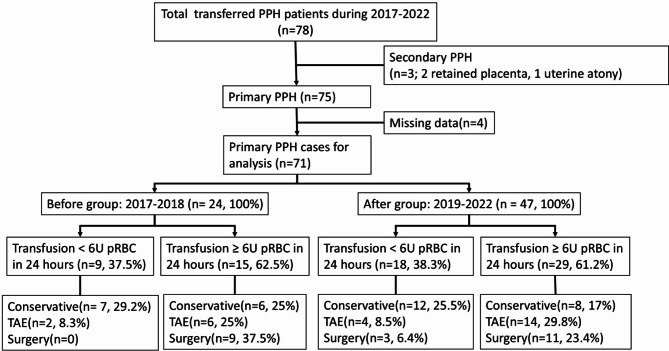
Patient selection and management flowchart. Flow diagram showing patient selection and distribution. From a total of 78 transferred PPH patients (2017–2022), 71 primary PPH cases were included for analysis after excluding 3 secondary PPH cases and 4 cases with missing data. Cases were divided into before (2017–2018, *n* = 24) and after (2019–2022, *n* = 47) groups, then further categorized by transfusion amount and management approach

During the study period (2017–2022), 78 patients with PPH were transferred to our emergency department. Seven patients were excluded: three due to secondary PPH and four due to incomplete data, leaving 71 patients for final analysis (Fig. 2). Among these, 24 patients were categorized into the BG, and 47 into the AG.

Significant bleeding, defined as the need for blood transfusion exceeding 6 units of packed RBC within 24 h, was observed in 15 patients (62.5%) in the BG and 29 patients (61.7%) in the AG. Treatment modalities included conservative management, TAE, and surgical intervention, as detailed in Fig. 2.

**Fig. 3:**
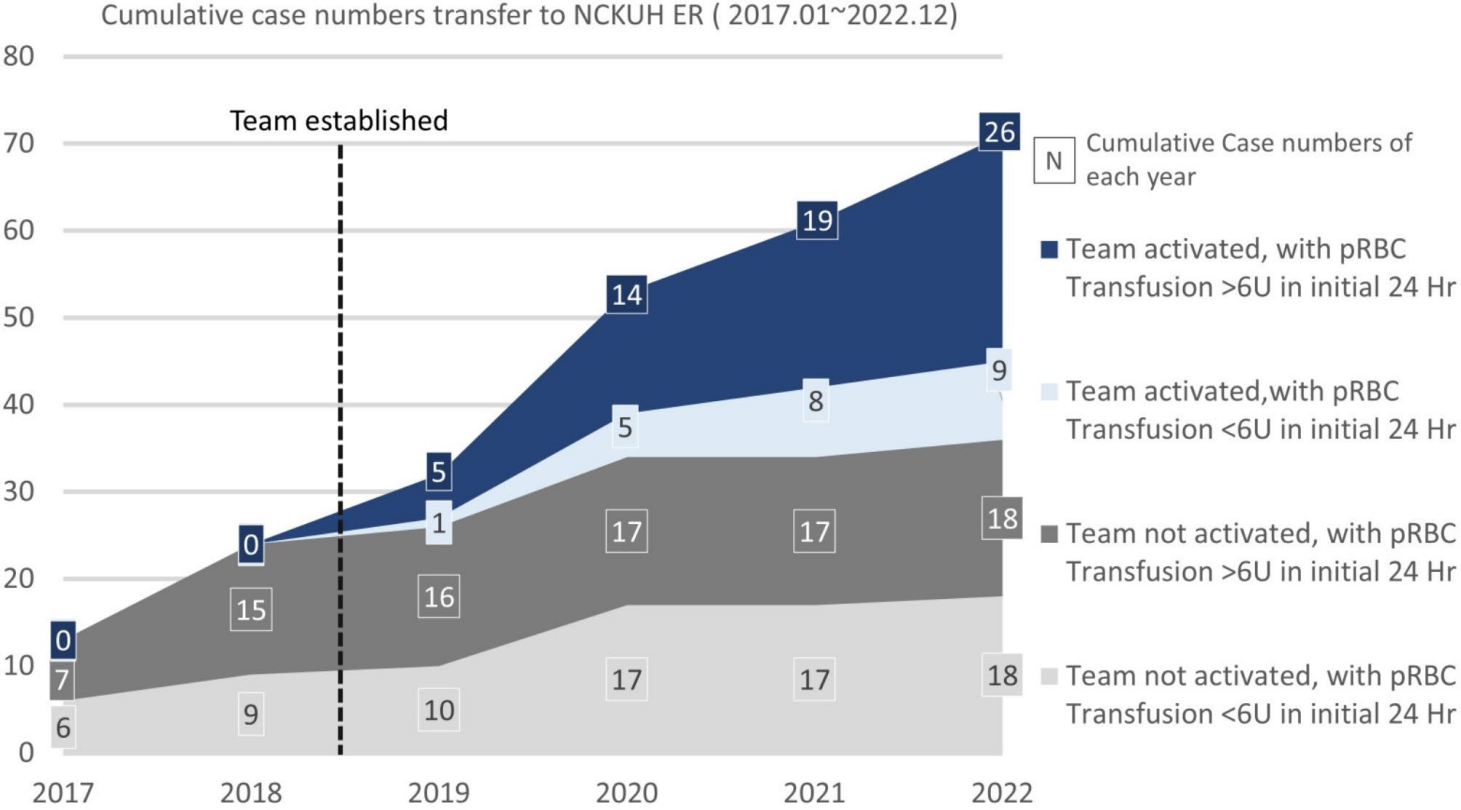
The number of patients transferred to the National Cheng Kung University Hospital emergency department during the study period. The multidisciplinary team was established and became operational in February 2019. The figure illustrates cumulative cases of postpartum hemorrhage (PPH) transferred to the NCKUH ER from 2017 to 2022, categorized by whether the team was activated. Cases where the team was activated are shown in blue, while those where the team was not activated are shown in gray. Among the twelve cases without team activation after February 2019, nine involved blood transfusions of less than six units within 24 h (light gray), indicating minimal bleeding. In contrast, the majority of cases with significant bleeding (requiring more than six units of blood transfusion) resulted in team activation

The PPH team was activated in 36 cases (76.6%) in the AG, with only three patients with significant bleeding not receiving team activation. Team activation rates demonstrated an increasing trend over the study period (Fig. 3).

The BG and AG were comparable in terms of demographic characteristics (age, gravidity, and parity), PPH aetiology, and delivery mode. Initial shock severity parameters, including shock index and base excess, and PPH management strategies were also similar between the groups (Table 1).

### Process-related quality indicators

Compared to the BG, the AG demonstrated significant improvements in several process-related quality indicators. These included a higher rate of tranexamic acid administration in the emergency room (74.47% vs. 33.33%, *P* = 0.002), a shorter time to initial blood transfusion (8 vs. 11 min, *P* = 0.029), and an increased proportion of patients reaching the OR within 60 min (80% vs. 25%, *P* = 0.014).

Subgroup analysis of patients with significant bleeding revealed similar improvements. During the first hour after emergency room arrival, while red blood cell transfusion volumes were comparable between groups (8 vs. 6 units, *P* = 0.114), the AG showed a significantly reduced requirement for fresh frozen plasma transfusions (6 vs. 4 units, *P* = 0.033). Other parameters, including the use of large-bore central venous catheters, activation of the massive transfusion protocol, and time to the angiography room, remained similar between the groups in both the overall and subgroup analyses (Table 2).

### Clinical outcomes

Clinical outcomes demonstrated significant improvements in the AG. Compared to the BG, the AG showed a shorter ICU length of stay (4 vs. 1 days, *P* = 0.005) and a lower incidence of coagulopathy (PT INR > 1.5) upon ICU admission (50% vs. 0%, *P* = 0.01). The incidence of hypothermia and acidosis was comparable between groups. Other clinical parameters, including hospital length of stay, ICU admission rate, duration of mechanical ventilation, hysterectomy rate, and mortality, remained similar between groups. Subgroup analysis yielded consistent results (Table 3).

Compared with the BG, the AG required fewer CPR procedures (4, 16.67% vs. 0, 0%, *P* = 0.011), had a lower proportion of patients receiving RBC transfusion ≥ 24 units within 24 h (5, 21.74% vs. 0, 0%, *P* = 0.022), and a lower proportion of patients requiring TAE after surgical intervention (4, 16.67% vs. 1, 2.13%, *P* = 0.042).

In the current study, there was one mortality (1/71, 1.4%), which occurred in the BG (4.17%) in the emergency room (ER), with no mortalities reported in the AG (0%). The difference was not statistically significant (*p* = 0.341). (Table 1).

## Discussion

A multidisciplinary team is vital for the life-saving management of critical PPH patients [8–10]. However, there is limited guidance on the practical establishment of such an efficient multidisciplinary team. In this study, we demonstrated a rapid and effective approach to establishing a life-saving PPH team by leveraging trauma management experience. The implementation of the multidisciplinary team activation system significantly improved both process-related quality indicators and clinical outcomes. For instance, the cardiac arrest rate decreased from 16.67 to 0%, and ICU length of stay was reduced from four days to one day after the team’s establishment.

Patients with PPH transferred to tertiary hospitals frequently present with severe hemodynamic instability due to uncontrolled bleeding and the risks associated with transportation [5, 13, 25]. Immediate and aggressive resuscitation is essential to salvage these critically ill patients, with early haemostasis being paramount for their survival [13, 25]. Umeda et al. demonstrated that the establishment of a rapid-response multidisciplinary team significantly improved the quality of care and clinical outcomes for PPH patients transferred to a tertiary emergency centre [13]. Our findings align with these results, showing significant improvements in multiple process-related quality indicators following the implementation of a structured multidisciplinary response system. This system effectively coordinated all team members, leading to notable reductions in CPR rates, transfusion times, OR times, blood transfusion volumes, coagulopathy, acidosis, salvage procedures, and ICU stays.

Previous studies have highlighted that adopting a protocol-based approach with a care bundle for PPH management enhances patient safety, reduces blood product usage, and decreases the incidence of postpartum hysterectomy [26, 27]. In 2023, a randomised trial demonstrated improved identification of PPH and enhanced composite outcomes, including reduced surgery rates and mortality due to bleeding [7]. Our study corroborates these findings, indicating that meticulous and coordinated multidisciplinary cooperation, coupled with an aggressive resuscitation bundle, can effectively improve clinical outcomes for unstable PPH patients.

Simulation is recommended for improving team communication, skills, and patient safety [17]. Studies have demonstrated positive outcomes and reduced blood transfusion rates following simulation training [18, 28]. However, simulation requires substantial resources and time to complete the training [16, 17]. Additionally, not all hospitals have access to a simulation centre. In our approach, we leveraged the existing trauma team’s experience, eliminating the need for specialized training. Emergency room physicians, interventional radiologists, and anesthesiologists had already developed proficiency in managing unstable bleeding trauma patients through collaborative protocols. Their established interdisciplinary communication and response mechanisms transferred effectively to PPH management, enabling immediate operational effectiveness. The team’s pre-existing expertise in critical bleeding management facilitated rapid adaptation to the PPH protocol. By building upon this well-practiced trauma team collaboration, we bypassed the need for extensive training and quickly established an efficient multidisciplinary response team. Our study demonstrates an alternative, practical approach to establishing a multidisciplinary team for PPH patients.

Previous studies have reported similar findings. Danial et al. demonstrated the successful formation of a rapid-response obstetrics team modelled after the cardiac arrest team, which resulted in improved mortality outcomes for patients with major obstetric haemorrhage, particularly those with placenta previa. Leveraging the experience of other types of emergency teams has proven to be an effective approach to treat PPH [24].

Our findings emphasise the positive impact of this multidisciplinary team activation system on PPH management and patient outcomes. By adopting recommendations and experience from our trauma team, we established effective team collaboration patterns and protocols for the PPH team without requiring trauma surgeons’ direct participation. The team was able to function effectively within a short period. The model and workflow are replicable, enabling the rapid establishment of a functional multidisciplinary PPH team even without simulation training. This multidisciplinary approach enhanced care quality and clinical outcomes within a short timeframe (Fig. 3), showing significant improvements even without the direct involvement of trauma surgeons. This method offers a practical solution for establishing an efficient multidisciplinary PPH team, particularly in facilities with experienced trauma teams.

### Strengths

Our strength lies in being among the few studies to explore the establishment of a multidisciplinary team [22, 24]. We provide a detailed description of the differences in processes before and after the establishment of the multidisciplinary team and collect both process-related quality indicators and patient outcomes. From the perspectives of process efficiency and clinical outcomes, we have demonstrated that leveraging trauma team experience can facilitate the establishment of an efficient multidisciplinary team within a short timeframe. This study offers a straightforward, effective, and practical approach for establishing such a team, which, to the best of our knowledge, has not been previously proposed.

### Limitations

This study has several limitations. First, the retrospective design introduces potential biases, including selection bias and chronological bias. The effect of chronological bias was minimised by the relatively short six-year study period and the consistency of general PPH treatment algorithms during this time. Second, although conducted at a tertiary care centre, the sample size was small. Nevertheless, despite the limited sample size, our study demonstrated significant improvements in quality. Third, as this is a retrospective study, accurate data regarding resuscitation timing, detailed management, initial blood loss, and blood transfusions administered at the original facility were unavailable. These limitations may have led to an underestimation of the actual blood transfusion volume, the initial severity of patient conditions, and any delays in treatment. The future implementation of a registration-based system may help overcome these challenges and enhance data accuracy.

**Table 1:** Demographic characteristics of patients

Variable	Overall			RBC Transfusion ≥6 units in 24 h		
	Before team established (*N* = 24)	After team established (*N* = 47)	*P* value	Before team established (*N* = 15)	After team established (*N* = 29)	*P* value
	*N* (%) / Median (IQR)	*N* (%) / Median (IQR)		*N* (%) / Median (IQR)	*N* (%) / Median (IQR)	
Age	34.0 (31.5, 37.0)	35.0 (32.0, 38.0)	0.476	34.0 (31.0, 36.0)	35.0 (32.0, 38.0)	0.519
Gravida						
1	12 (50.00)	12 (25.53)	0.094	6 (40.00)	6 (20.69)	0.158
2	8 (33.33)	19 (40.43)		7 (46.67)	11 (37.93)	
>3	4 (16.67)	16 (34.04)		2 (13.33)	12 (41.38)	
Para						
1	14 (58.33)	19 (40.43)	0.357	7 (46.67)	11 (37.93)	0.563
2	7 (29.17)	19 (40.43)		6 (40.00)	10 (34.48)	
>3	3 (12.50)	9 (19.15)		2 (13.33)	8 (27.59)	
Delivery						
C/S	18 (75.00)	34 (72.34)	1.000	12 (80.00)	19 (65.52)	0.488
VD	6 (25.00)	13 (27.66)		3 (20.00)	10 (34.48)	
**Etiology**						
Tissue^#^	2 (8.33)	6 (12.77)	0.708	1 (6.67)	4 (13.79)	0.647
Trauma^$^	16 (66.67)	29 (61.70)	0.881	11 (73.33)	21 (72.41)	1.000
Atony	9 (37.50)	20 (42.55)	0.877	5 (33.33)	11 (37.93)	1.000
Coagulopathy	4 (16.67)	4 (8.51)	0.430	4 (26.67)	3 (10.34)	0.207
**Severity**						
Shock index						
<0.9	8 (36.36)	20 (42.55)	0.844	3 (23.08)	8 (27.59)	1.000
0.9 to < 1.2	9 (40.91)	16 (34.04)		5 (38.46)	10 (34.48)	
≥1.2	5 (22.73)	11 (23.40)		5 (38.46)	11 (37.93)	
BE (mEq/L)						
−2 to 0	1 (4.76)	1 (2.33)	0.616	1 (7.69)	0 (0.00)	0.206
≥−6 to < − 2	5 (23.81)	14 (32.56)		2 (15.38)	7 (25.00)	
≥−10 to < − 6	10 (47.62)	22 (51.16)		5 (38.46)	16 (57.14)	
<−10	5 (23.81)	6 (13.95)		5 (38.46)	5 (17.86)	
Fibrinogen < 200(mg/dL)	4 (25.00)	7 (21.88)	1.000	4 (44.44)	6 (26.09)	0.407
Initial management (< 6 h)						
Conservative treatment	13 (54.17)	20 (42.55)	0.650	6 (40.00)	8 (27.59)	0.667
Operation	4 (16.67)	10 (21.28)		4 (26.67)	8 (27.59)	
TAE	7 (29.17)	17 (36.17)		5 (33.33)	13 (44.83)	
Salvage procedure						
Operation after TAE	2 (8.33)	4 (8.51)	1.000	2 (13.33)	3 (10.34)	1.000
TAE after operation	4 (16.67)	1 (2.13)	0.042^*^	4 (26.67)	1 (3.45)	0.039^*^
Intervention after 6 h						
Operation	1 (4.17)	0 (0.00)	0.111	1 (4.17)	0 (0.00)	0.341
TAE	1 (4.17)	0 (0.00)		0 (0.00)	0 (0.00)	
Non	22 (91.67)	47 (100.00)		14 (93.33)	29 (100.00)	
RBC in 24 h						
<6 units	8 (34.78)	18 (38.30)	0.022^*^	0 (0.00)	0 (0.00)	0.010^*^
6 to < 12 units	5 (21.74)	18 (38.30)		5 (33.33)	18 (62.07)	
12 to < 18 units	4 (17.39)	8 (17.02)		4 (26.67)	8 (27.59)	
18 to < 24 units	1 (4.35)	3 (6.38)		1 (6.67)	3 (10.34)	
≥24 units	5 (21.74)	0 (0.00)		5 (33.33)	0 (0.00)	
CPR	4 (16.67)	0 (0.00)	0.011^*^	4 (26.67)	0 (0.00)	0.010^*^

*C/S*, Cesarean section; *VD*, vaginal delivery; *BE*, base excess; *TAE*, transcatheter arterial embolization; *RBC*, packed red blood cells; *CPR*, cardiopulmonary resuscitation. *: significant difference; ^#^Tissue, defined as retained placental tissues; ^$^Trauma, defined as genital tract or uterine laceration which was not repaired;

**Table 2:** Process-related quality indicators

Variable	Overall			RBC Transfusion ≥6 units in 24 h		
	Before team established (*N* = 24)	After team established (*N* = 47)	*P* value	Before team established (*N* = 15)	After team established (*N* = 29)	*P* value
	*N* (%) / Median (IQR)	*N* (%) / Median (IQR)		*N* (%) / Median (IQR)	*N* (%) / Median (IQR)	
Tranexamic acid in ER	8 (33.33)	35 (74.47)	0.002^*^	4 (26.67)	22 (75.86)	0.005^*^
Time to first transfusion in ER(min)	11.00 (8.00, 21.00)	8.00 (0.00, 13.00)	0.029^*^	9.00 (8.00, 14.00)	6.00 (0.00, 12.00)	0.054
RBC in first hour	4.00 (2.00, 12.00)	4.00 (2.00, 6.00)	0.694	8.00 (4.00, 16.00)	6.00 (4.00, 8.00)	0.114
FFP in first hour	4.00 (0.00, 10.00)	2.00 (0.00, 4.00)	0.069	6.00 (4.00, 12.00)	4.00 (2.00, 6.00)	0.033^*^
Large-bore CVC	8 (33.33)	17 (36.17)	1.000	6 (40.00)	16 (55.17)	0.525
MTP	6 (25.00)	10 (21.28)	0.956	6 (40.00)	9 (31.03)	0.800
Time to first initial intervention	*n* = 11	*n* = 27		*n* = 9	*n* = 21	
≤30 min	0 (0.00)	1 (3.70)	0.808	0 (0.00)	1 (4.76)	1.000
>30 to ≤60 min	6 (54.55)	11 (40.74)		4 (44.44)	9 (42.86)	
>60 to ≤120 min	4 (36.36)	9 (33.33)		4 (44.44)	7 (33.33)	
>120 min	1 (9.09)	6 (22.22)		1 (11.11)	4 (19.05)	
Time to OR	*n* = 4	*n* = 10		*n* = 4	*n* = 8	
≤30 min	0 (0.00)	0 (0.00)	0.014^*^	0 (0.00)	0 (0.00)	0.018^*^
>30 to ≤60 min	1 (25.00)	8 (80.00)		1 (25.00)	7 (87.50)	
>60 to ≤120 min	3 (75.00)	0 (0.00)		3 (75.00)	0 (0.00)	
>120 min	0 (0.00)	2 (20.00)		0 (0.00)	1 (12.50)	
Time to angiography room	*n* = 7	*n* = 17		*n* = 5	*n* = 13	
≤30 min	0 (0.00)	1 (5.88)	0.090	0 (0.00)	1 (7.69)	0.374
>30 to ≤60 min	5 (71.43)	3 (17.65)		3 (60.00)	2 (15.38)	
>60 to ≤120 min	1 (14.29)	9 (52.94)		1 (20.00)	7 (53.85)	
>120 min	1 (14.29)	4 (23.53)		1 (20.00)	3 (23.08)	
TAE in OR	2 (8.33)	0 (0.00)	0.111	2 (13.33)	0 (0.00)	0.111

*RBC*, packed red blood cells; *FFP*, fresh frozen plasma; *CVC*, central venous catheter; *MTP*, massive blood transfusion; *OR*, operating room; *TAE*, transcatheter arterial embolization. *: significant difference

**Table 3:** Clinical outcomes

Variable	Overall			RBC Transfusion ≥6 units in 24 h		
	Before team established (*N* = 24)	After team established (*N* = 47)	*P* value	Before team established (*N* = 15)	After team established (*N* = 29)	*P* value
	*N* (%) / Median (IQR)	*N* (%) / Median (IQR)		*N* (%) / Median (IQR)	*N* (%) / Median (IQR)	
LOS	3.00 (2.00, 5.00)	3.00 (2.00, 4.00)	0.757	3.00 (2.00, 8.00)	4.00 (3.00, 4.00)	0.920
ICU admission	8 (33.33)	14 (29.79)	0.973	8 (53.33)	14 (48.28)	1.000
Days in ICU	4.00 (2.00, 5.00)	1.00 (1.00, 2.00)	0.005^*^	4.00 (2.00, 5.00)	1.00 (1.00, 2.00)	0.005^*^
Initial ICU BT < 35’C	2 (25.00)	0 (0.00)	0.121	2 (25.00)	0 (0.00)	0.121
Initial ICU lactate > 4.0(mmol/L)	7 (87.50)	6 (46.15)	0.085	7 (87.50)	6 (46.15)	0.085
Initial ICU PT(INR) > 1.5	4 (50.00)	0 (0.00)	0.010	4 (50.00)	0 (0.00)	0.010
Initial ICU BE(mEq/L)						
−2 to 0	2 (33.33)	4 (28.57)	1.000	2 (33.33)	4 (28.57)	1.000
−6 to < − 2	2 (33.33)	6 (42.86)		2 (33.33)	6 (42.86)	
−10 to < − 6	1 (16.67)	2 (14.29)		1 (16.67)	2 (14.29)	
<−10	1 (16.67)	2 (14.29)		1 (16.67)	2 (14.29)	
Days on ventilator	*n* = 6 / 2.00 (1.00, 4.00)	*n* = 7 / 1.00 (1.00, 2.00)	0.586	*n* = 6 / 2.00 (1.00, 4.00)	*n* = 7 / 1.00 (1.00, 2.00)	0.586
Hysterectomy	3 (12.50)	3 (6.38)	0.399	3 (20.00)	3 (10.34)	0.394
Mortality	1 (4.17)	0 (0.00)	0.338	1 (6.67)	0 (0.00)	0.341

*LOS*, length of stay; *BT*, body temperature; *PT (INR)*, prothrombin time (international normalized ratio). *: significant difference

## Conclusion

**Fig. 4:**
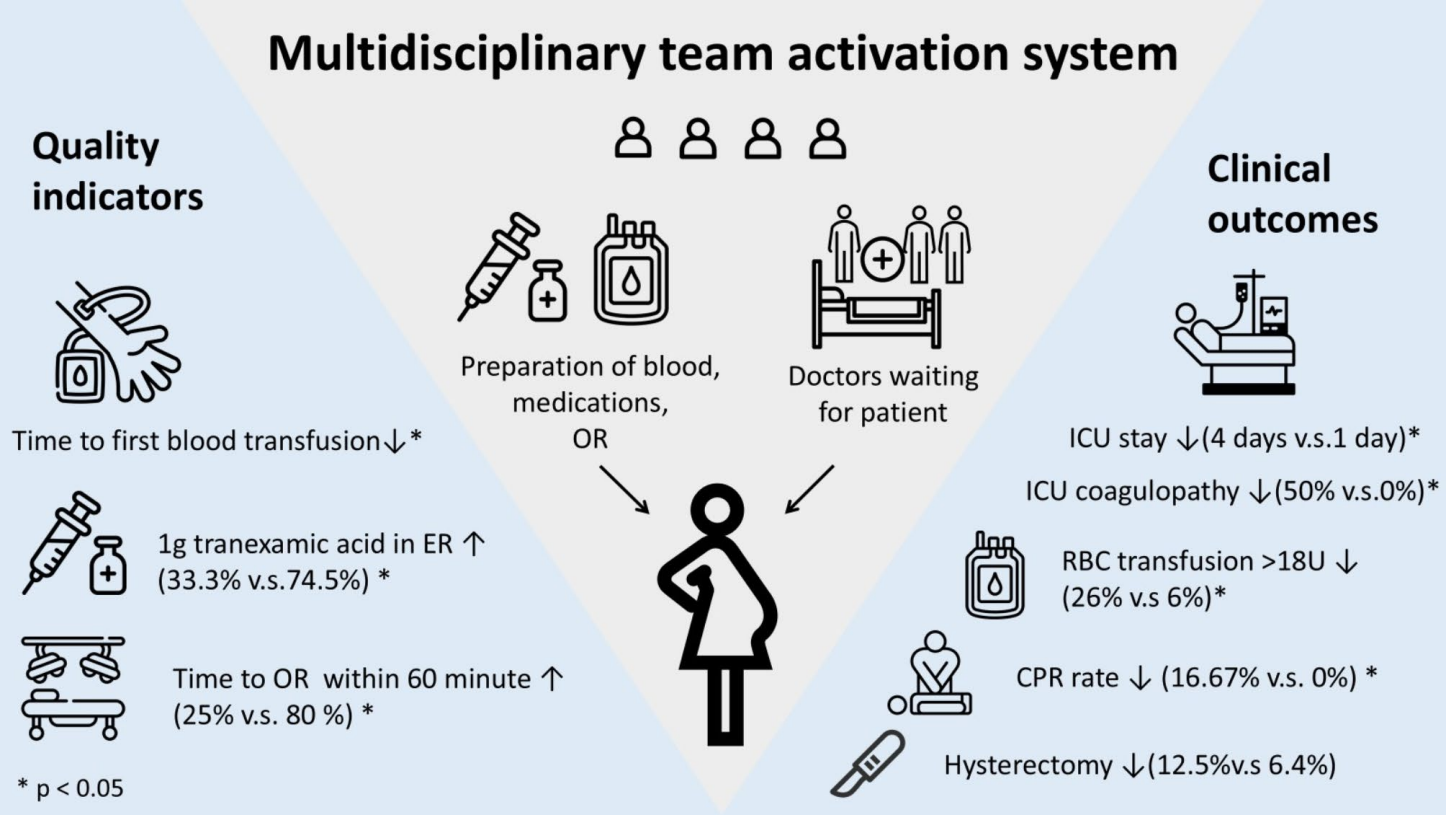
Geographical abstract of the study result

We proposed a method for establishing a multidisciplinary PPH team tailored to the existing system by leveraging trauma team experience. This newly established multidisciplinary PPH team was supported by pre-arrival ER preparation and significantly improved both the quality of patient management and clinical outcomes (Fig. 4). For hospitals with a mature trauma system, this study provides an efficient and feasible approach to establishing a multidisciplinary PPH team to reduce morbidity and mortality for critically ill postpartum patients.

## Data Availability

The datasets used and analysed during the current study are available from the corresponding author on reasonable request.

## Abbreviations

PPH: Postpartum Hemorrhage
NCKUH: National Cheng Kung University Hospital
ER: Emergency Room
OR: Operating Room
TAE: Transcatheter Arterial Embolization
FFP: Fresh Frozen Plasma
pRBC: Packed Red Blood Cells
CPR: Cardiopulmonary Resuscitation
ICU: Intensive Care Unit
PT INR: Prothrombin Time International Normalized Ratio
